# The Role of Endovascular Procedure for Peripheral Arterial Disease in Diabetic Patients With Chronic Limb-Threatening Ischemia

**DOI:** 10.7759/cureus.23857

**Published:** 2022-04-05

**Authors:** Yudistira Santosa, Azizah Dhena Harca, Edwin Sukmaja, Angelina Yuwono

**Affiliations:** 1 Department of Internal Medicine, School of Medicine and Health Sciences, Atma Jaya Catholic University of Indonesia, Jakarta, IDN; 2 General Medicine, Primaya Hospital, Jakarta, IDN

**Keywords:** diabetic foot, chronic limb threatening ischemia, peripheral arterial disease, diabetes mellitus, endovascular

## Abstract

Type 2 diabetes mellitus is a major risk factor for all forms of cardiovascular diseases, including peripheral arterial disease (PAD). Chronic limb-threatening ischemia (CLTI) is determined by the presence of ischemic rest pain, and may or may not be accompanied by tissue loss (such as ulcers and gangrene) or infection. Treatments for CLTI consist of wound treatment, infection control, and ischemia control by arterial revascularization, which can be performed by either open surgical procedure (bypass) or an endovascular approach. We present two cases of chronic limb-threatening ischemia, one with an above-knee lesion and the other with a below-knee lesion. In addition to good wound treatment and glucose control as the risk factor management, we performed endovascular therapy in both patients. Both patients showed good wound improvement after the procedure.

## Introduction

Peripheral arterial disease (PAD), a well-known macrovascular complication of diabetes mellitus, affects about 236 million adults worldwide and is more prevalent in high-income than in low-income countries [1]. A study in Indonesia, conducted by Ismail et al., reported that PAD prevalence in type 2 diabetes mellitus (T2DM) patients is 16% [2]. Atherosclerosis is the most common cause of PAD. This disease may affect any artery and is commonly found in the lower extremities. One severe form of PAD is chronic limb threatening ischemia (CLTI), characterized by ischemic rest pain, tissue loss, or gangrene, which are present for more than two weeks. CLTI is estimated to affect 1.3% of individuals aged 40 years or older. Of all patients with PAD, 11% experienced CLTI [1].

In a patient with PAD and foot ulcer, wound healing should be estimated based on clinical examination as well as non-invasive vascular evaluations. Many factors may disturb the wound healing process, such as infection, necrotic tissue, poor blood glucose control, comorbidities, and abnormal mechanical loading of the ulcer [3]. PAD is responsible for increasing the risk of non-traumatic lower extremity amputation, especially in diabetic patients [1].

Revascularization should always be considered in patients who experienced persistent ischemic rest pain or those who have a low probability of wound healing, because of the decreased blood flow that occurs in PAD [4]. If revascularization is not done, most CLTI will result in limb loss [5]. Arterial revascularization is either performed through an open procedure (such as a bypass procedure) or an endovascular approach using balloon dilatation and, if needed, an adjunctive stent. Although bypass surgery was chosen as the first choice to treat PAD, in the last decade, the revascularization procedures have been shifted to the endovascular approach as the procedure has been rapidly expanded. Even though many developments have been made, neither procedure ensures freedom from restenosis [4,5].

## Case presentation

Case 1: proximal lower limb lesion

**Figure 1:**
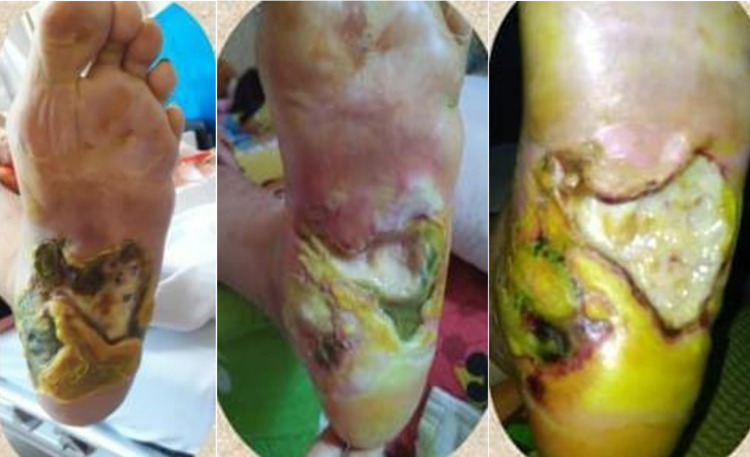
The wound on the distal left foot

A 59-year-old female with a history of 10 years of diabetes mellitus had suffered from a non-healing wound on her left foot for one year and she often felt pain in her left foot, which was accentuated by activity, such as walking. She reported a Visual Analogue Scale (VAS) score of 7/10. Her wound was regularly dressed, and she took oral diabetic agents to control her blood glucose. Despite her regular wound care, the wound didn’t heal and became more extensive with more purulent discharge and a foul odor. In physical examination, we found a grade III Wagner diabetic foot with the size of 8 x 10 cm, which had an irregular border, necrosis tissue, and exposed muscle with foul odor discharge (Figure 1).

**Figure 2:**
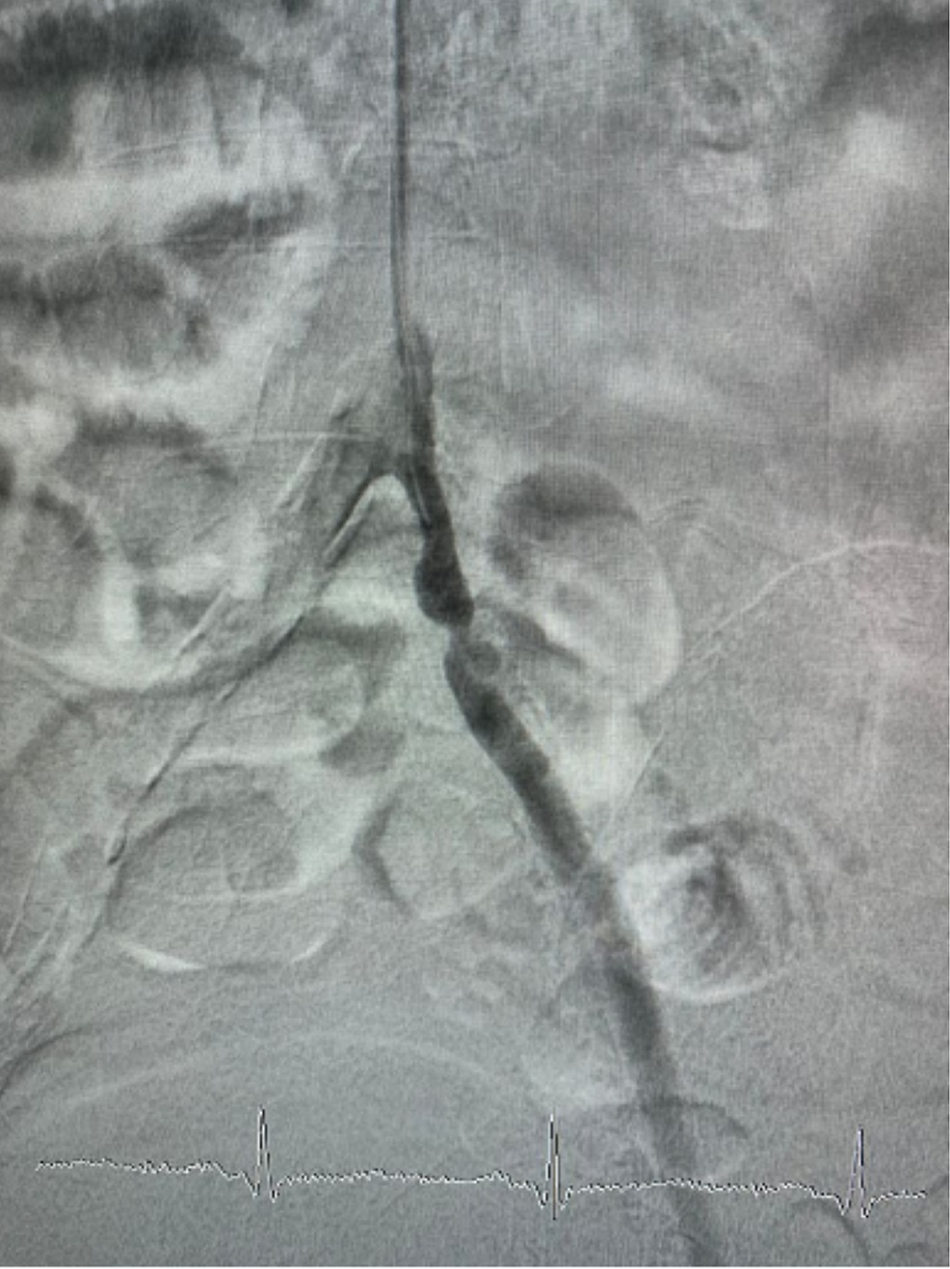
Arteriography in the left leg

Her left dorsalis pedis artery pulse was palpated weaker than its contralateral. Doppler sonography showed reduced perfusion from the left superficial femoral artery. Arteriography and percutaneous transcatheter angioplasty were performed, simultaneously with wound care, adequate antibiotic, and adequate blood glucose control. Arteriography showed 95% stenosis in the osteal left iliac artery (Figure 2). 

Because of the lesion in the osteal iliac artery, angioplasty was done with a double puncture from the right femoral artery and left brachial artery (Videos 1, 2, 3).

**Video 1:** Left brachial approach descent to the iliac artery

**Video 2:** Left brachial approach with left femoral artery approach

**Video 3:** Stent placement in osteal left Iliac artery

**Figure 3:**
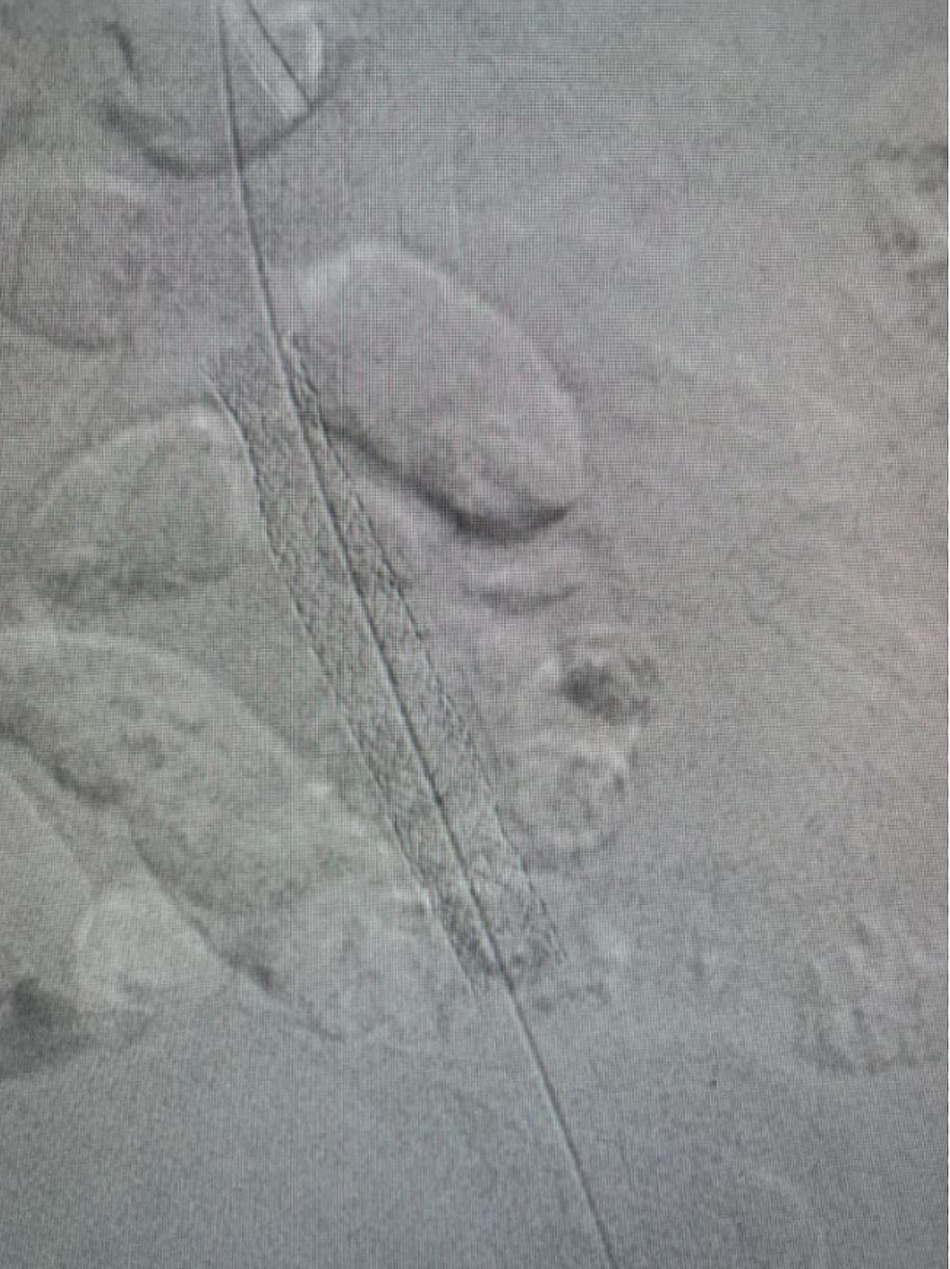
Stent in osteal left Iliac artery

**Figure 4:**
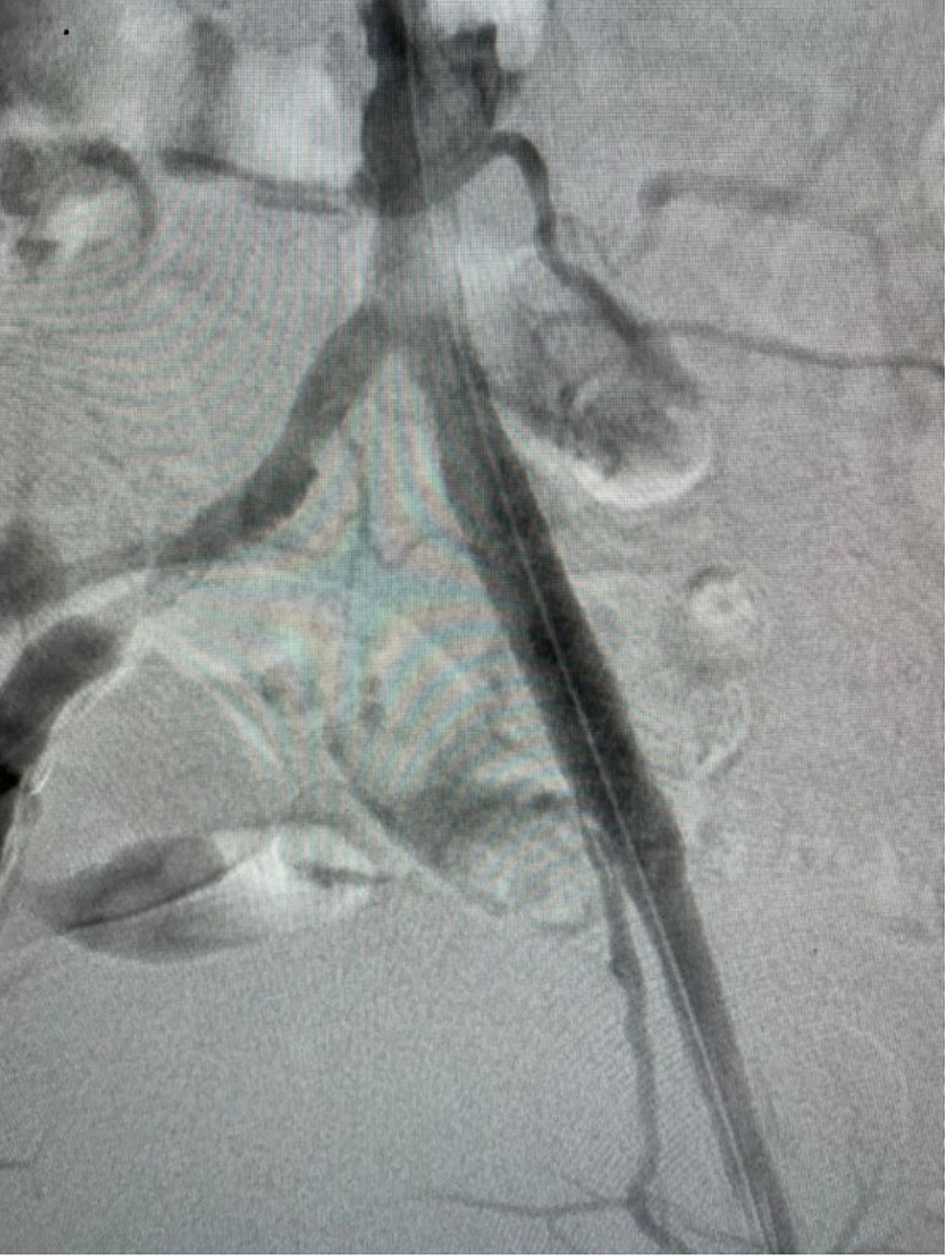
Result of endovascular treatment

**Figure 5:**
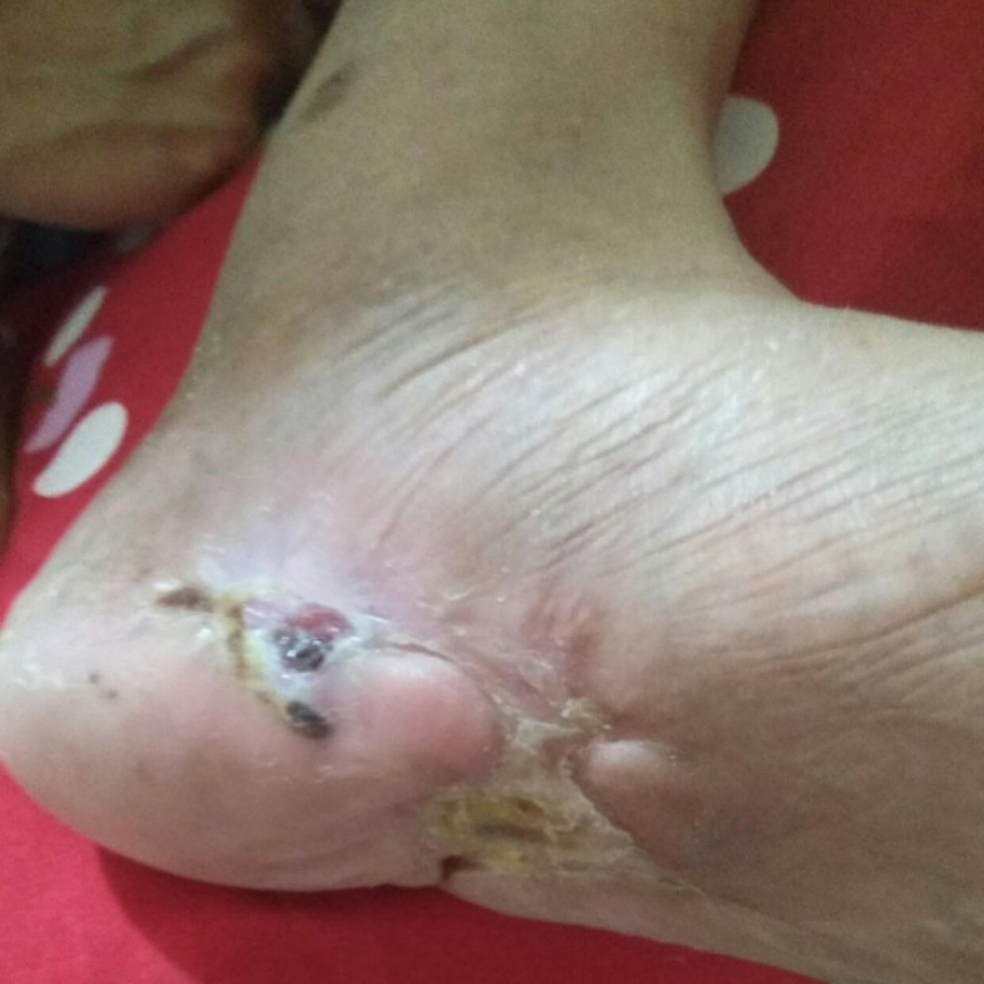
After two months angioplasty

After predilating the stenosis with a 3.0 mm balloon, a balloon-expandable bare metal stent of 7.0 mm was deployed in the left osteal iliac artery. The stent was fully expanded in the Iliac artery. The procedure was done without any complications (Video 4, Figures 3, 4). After the procedure, the wound showed a remarkable improvement and was completely healed within two months after angioplasty (Figure 5).

**Video 4:** Stent inflation in osteal left Iliac artery

Case 2: distal lower limb lesion

**Figure 6:**
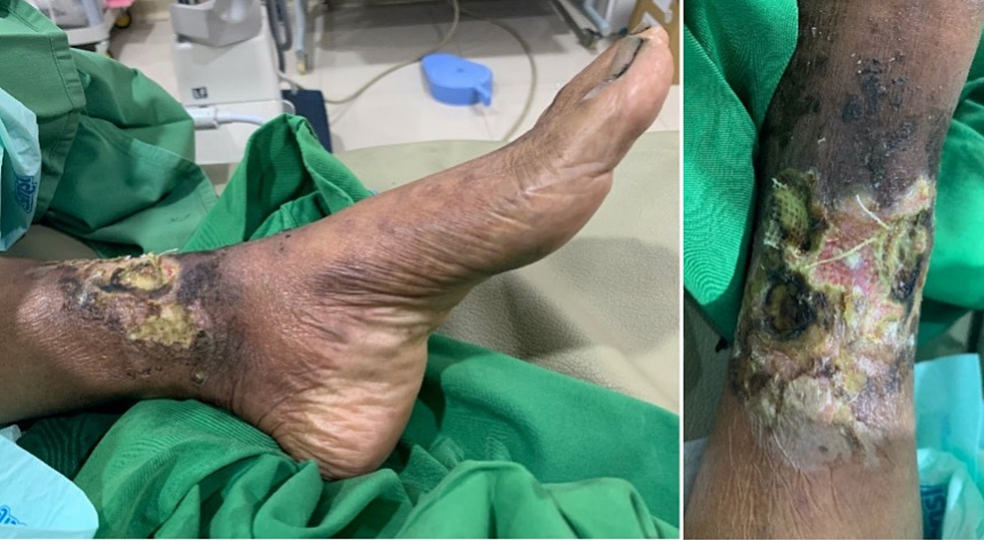
Diabetic left foot

A 47-year-old female with a history of five-year diabetes mellitus had suffered from a non-healing wound on her left foot for six months. She experienced rest pain below her knee (VAS 9/10), which immobilized her. Her distal foot was also numb. She was regularly treated with wound dressings and glycemic control drugs. Despite regular care, the wound progressed, and its discharge became more purulent with a foul odor. In physical examination, we found a grade IV (Fontaine classification) wound on her left foot. The wound had an irregular border, necrosis tissue, and exposed muscle with a foul odor discharge (Figure 6).

**Figure 7:**
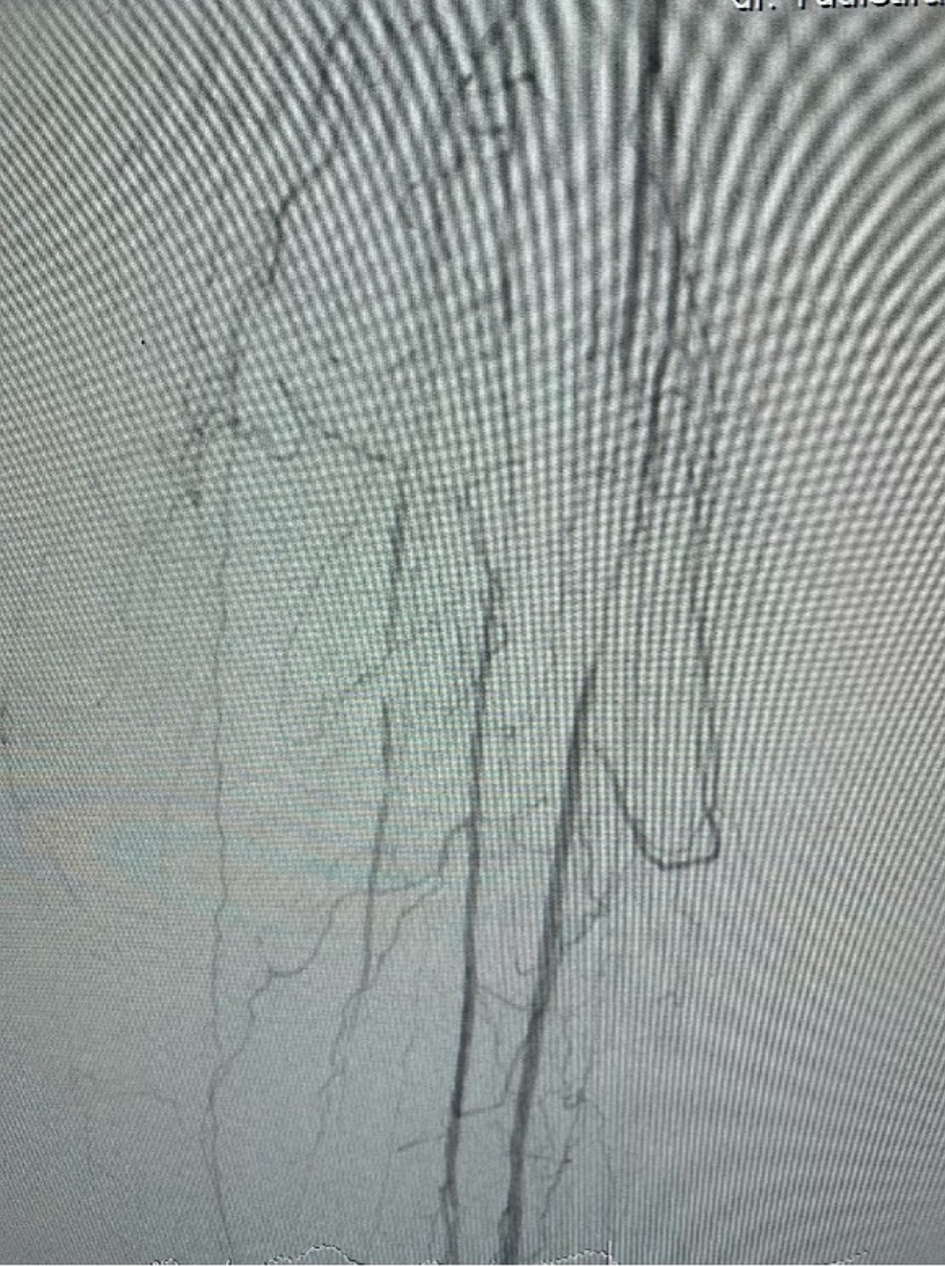
Arteriogram left leg (below knee)

From doppler ultrasound, we found monophasic flow in the popliteal artery and there was no flow in the medial segment anterior tibialis artery and medial segment posterior tibialis artery. She was then diagnosed with critical limb ischemia in a diabetic patient. We later did arteriography with a contralateral approach from the right femoral artery. Arteriogram showed total stenosis in the proximal anterior tibialis artery, peroneal artery, and posterior tibialis artery (Figure 7 and Video 5).

**Video 5:** Below Knee Arteriogram

**Figure 8:**
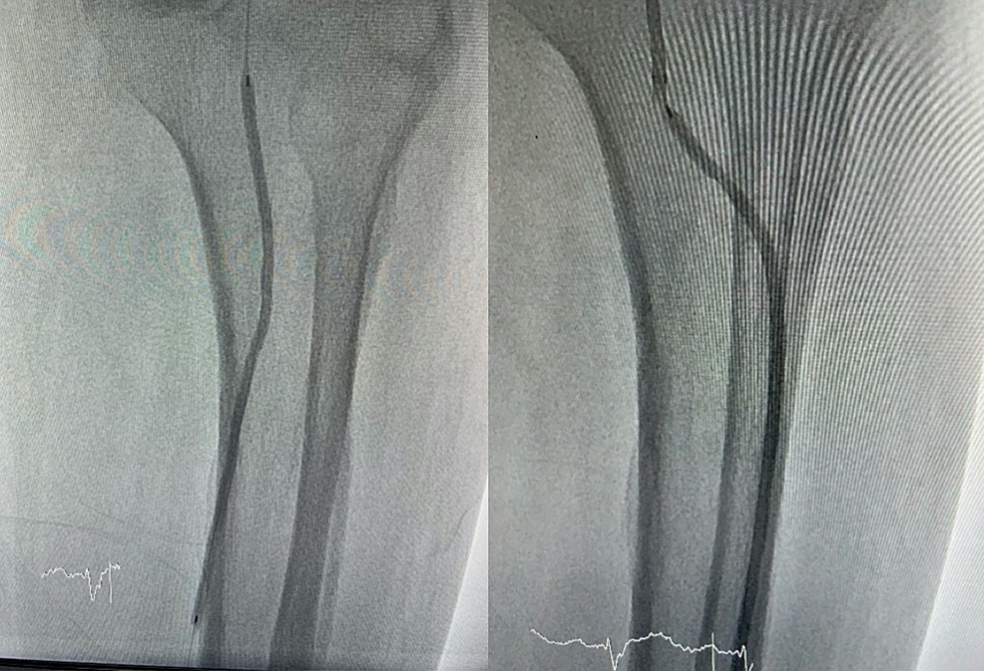
Endovascular treatment in the left leg

Endovascular treatment was done with an antegrade approach. We use JR 3.5 6 French guiding catheter with V14 guidewire. Lesions were dilated with a 3.0 mm drug-coated balloon. We dilated in the anterior tibialis artery and posterior tibialis artery (Figure 8, Videos 6, 7).

**Video 6:** Arteriogram after endovascular treatment (below the knee)

**Video 7:** Arteriogram after endovascular treatment (ankle)

**Figure 9:**
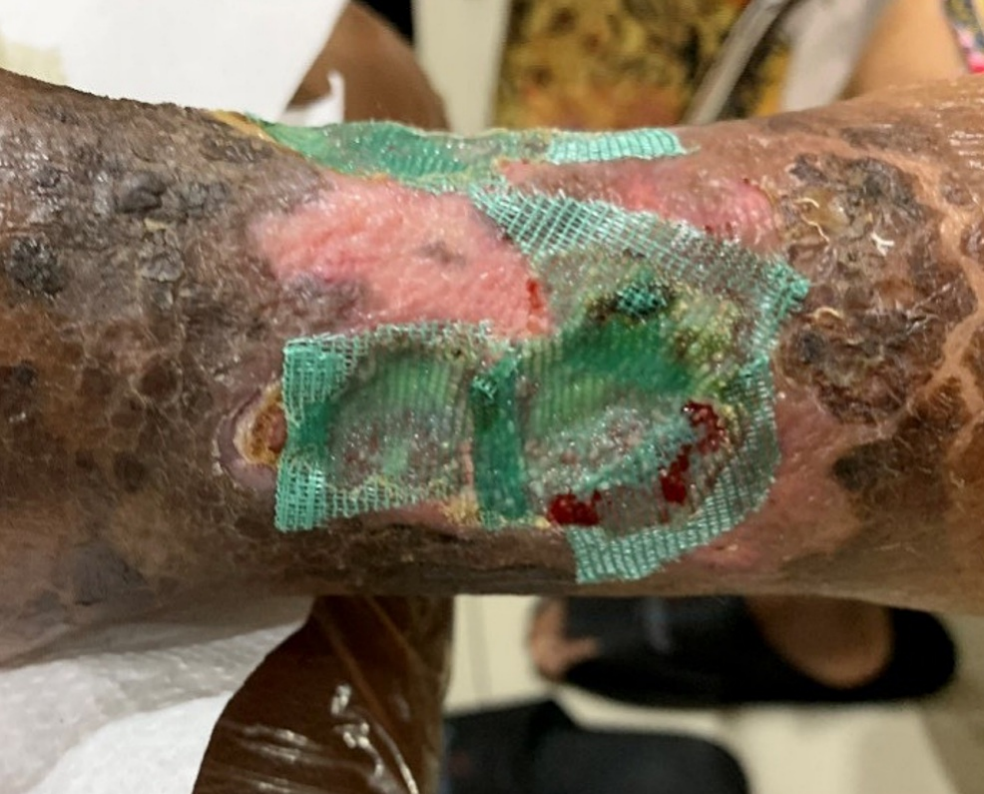
Wound after ten days of treatment

**Figure 10:**
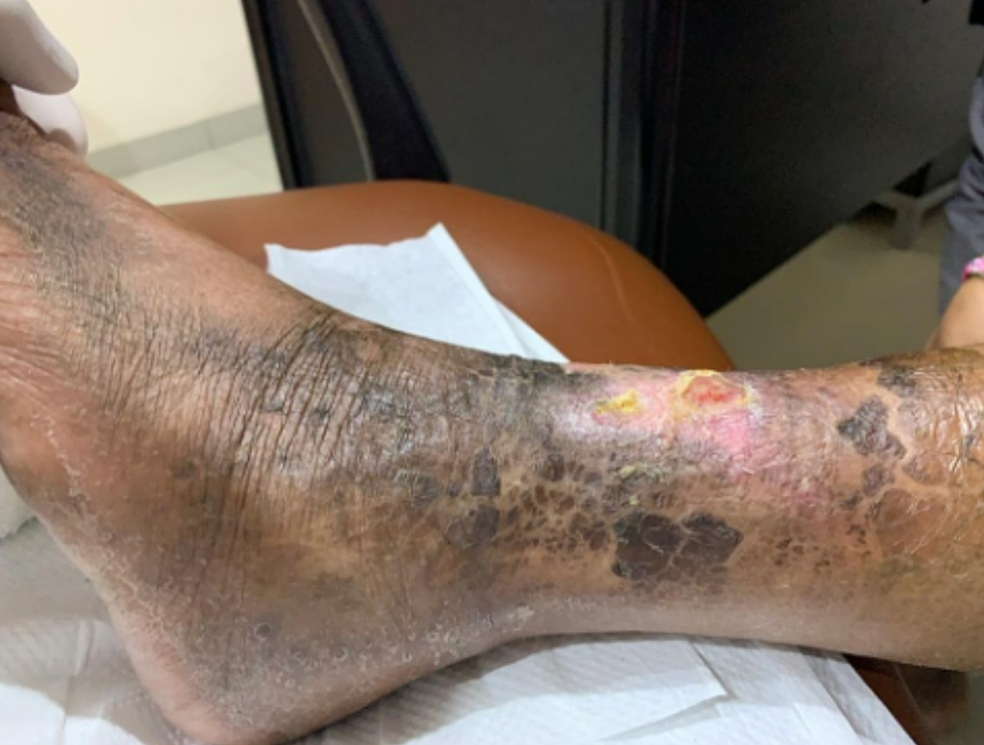
Wound after forty-five days of treatment

After endovascular treatment, the pain was reduced and the wound was gradually improved. After 10 days, the patient was able to walk normally. The wound was healed 45 days after treatment (Figures 9, 10).

## Discussion

Diabetes mellitus (DM) is already known as a major risk factor for cardiovascular diseases, which also account for the leading cause of death for adult diabetic patients. PAD is one of the macrovascular complications, and is defined as a partial complete obstruction in one or more peripheral arteries, such as in lower extremity arteries [6]. DM is also a strongest risk factor for PAD as it is occurs in 14% of diabetic patients with an odds ratio of 2.72. PAD is defined as a partial or complete obstruction in one or more peripheral arteries, such as in lower extremity arteries. Patients with DM and PAD have an increased risk of adverse cardiac and limb events and impaired quality of life. Moreover, PAD causes significant long-term disability in patients with DM [6]. Both of our patients had a long-standing history of DM before experiencing the wound. The first patient experienced intermittent claudication before having the wound and the second patient experienced numbness. These reflected the presence of PAD and neuropathy before having the wound.

Most patients have no symptoms and the disease is detected by a low Ankle Brachial Index (ABI) (less than <0.90) or diminished pulse, which is found on routine examination. ABI measurement is recommended in diabetic patients over 40 years old [7,8]. Many patients who have ABI less than 0.90 have severe obstruction, but experience no symptoms due to reduced pain sensitivity (as in diabetic neuropathy), or the incapability to walk for enough (as in heart failure) [7]. The most typical symptom of PAD is intermittent claudication, which was also experienced in our first patient. In diabetic patients, risk of claudication is increased by 8.6 fold in women and 3.5 men [8]. CLTI is defined by the presence of ischemic rest pain, with or without tissue loss (ulcers, gangrene) or infection, and these were also found in our second patient. Arterial ulcers are usually painful and are often complicated by local infection and inflammation [9]. The cardiovascular risk in PAD patients is stratified according to the severity of ischemia, the presence of infection, and the condition of the wounds. The risk is stratified according to the severity of ischemia, wounds, and infection. Diabetes is found in 50-70% of cases and it presents mostly as neuro-ischaemic diabetic foot ulcers [10].

Duplex ultrasound (DUS), combined with ABI measurement, is often chosen as the first work-up to screen and diagnose vascular diseases. Even though it is user dependent, DUS is a good non-invasive diagnostic method that may evaluate blood flow and gives no exposure to radiation. The DUS has B-mode echography, pulsed or continuous wave, with colour and power Doppler modalities, which play a role in detecting and localizing vascular lesions, as well as quantifying the extension and severity of PAD through the velocity criteria [10,11]. Other imaging modalities in diagnosing PAD are computed tomography angiography (CTA) or magnetic resonance angiography (MRA), and digital subtraction angiography (DSA) [10]. DUS was also done in both of our patients and determined the location of the occlusion, which was later used to guide the appropriate treatment.

Two important aspects in the management of PAD are symptom management and risk factor management. The first aspect of management is addressing specific symptoms related to the location of the lesion and its specific risk. The second aspect of management is pointed at the management of the risk of a cardiovascular event. CV prevention is warranted and should be managed by a multidiscipline team. The so-called "Best Medical Therapy" (BMT) includes the management of CV risk factors with both best pharmacological therapy and non-pharmacological management, such as smoking cessation, healthy diet, promoting weight loss, and regular physical exercise. The pharmacological therapy should include antihypertensive drugs, lipid-lowering agents, and anti-thrombotic drugs. Optimal blood glucose is mandatory in PAD patients who have diabetes [10].

Glycaemic control is particularly important to improve limb-related outcomes, which lowers the rate of major amputation and increases the patency after infra-popliteal revascularization. Wound care should be started immediately, as well as the use of adapted footwear, infection treatment and pain management. Revascularization should be attempted and the strategy should be depending on the complexity of the lesion. If an endovascular approach is attempted, landing zones for a potential bypass graft need to be preserved. If bypass surgery is taken, the bypass should be planned to be as short as possible and a saphenous vein is used. In CLTI, minor amputation may be necessary to remove necrotic tissues and may affect the patient's mobility. Revascularization should be done before the amputation to improve wound healing. Bypass surgery is considered in patients with long occlusion, who are fit for surgery, and who have a patent great saphenous vein. Surgical revascularization using a great saphenous vein bypass graft ensure better long-term patency. Endovascular therapy should be first attempted in patients with short occlusive stenotic lesions (lesser than 25 cm), long occlusions with no great saphenous vein, or those who have increased risk for open surgery. Studies comparing endovascular and surgical therapy are not available yet. Some new endovascular managements using atherectomy devices, drug-eluting balloons, or new stent designs have shown an improvement in long-term patency [10]. Both of our patients underwent revascularization procedures by endovascular treatment, which resulted in complete wound healing and symptoms resolution.

Endovascular therapy warrants good long-term patency (> 90% over five years) and low risk of complications in patients with isolated aorto-iliac lesions and short stenosis/occlusion (<5cm) of iliac arteries. A hybrid technique is considered in ileofemoral lesions. Endovascular therapy of iliac arteries is combined with endarterectomy or femoral bypass. Endovascular reconstruction of an aortic bifurcation is considered in those whose occlusion extends to the infrarenal aorta [10]. Long-term patency and stents durability are the challenges of endovascular therapy of the femoropopliteal region, in which the artery is very mobile [10]. For lesions below the knee level or the infrapopliteal lesion, it is unknown whether drug-eluting stents and drug-coated balloons give more benefits than the previously established technique using plain balloons and bare-metal stents [12,13]. Our first patient presented with a wound in her left plantar pedis and arteriography showed a 95% stenosis in the left osteal iliac artery. In this patient, we did an endovascular approach through the brachial and femoral arteries to place a stent in the iliac artery. The second patient showed a total stenosis in the proximal anterior tibialis artery, peroneal artery, and posterior tibialis artery, which are all located infrapopliteal. We placed a drug-coated balloon in her anterior and posterior tibialis artery. Both patients showed good resolution in pain and good wound healing process.

## Conclusions

Type 2 diabetes melitus is a risk factor for peripheral artery disease (PAD). Chronic limb-threatening ischemia (CLTI) is defined by the presence of ischaemic rest pain, with or without tissue loss or infection. Treatment of CLTI with arterial revascularization can be performed through open bypass surgery or an endovascular procedure and may result in a better outcome of wound healing.
